# Atypical Presentation of Pancreatic Adenocarcinoma as a Fever of Unknown Origin Mimicking Multiple Liver Abscesses: A Case Report

**DOI:** 10.7759/cureus.106413

**Published:** 2026-04-03

**Authors:** Yuvrajsing K Pakal, Neha P Dharap, Shreya N Gade

**Affiliations:** 1 Department of Medicine, Smt. Kashibai Navale Medical College and General Hospital, Pune, IND

**Keywords:** cancer metastasis, fever of unknown origin (fuo), pancreatic adenocarcinoma, pseudo liver abscess, serum tumor markers

## Abstract

Pancreatic adenocarcinoma typically presents late, most often with nonspecific symptoms such as weight loss, jaundice, and abdominal pain. Presentation with prolonged fever and weakness mimicking an infectious etiology is exceedingly rare. We report a case of a 54-year-old man with no known comorbidities who presented with persistent fever, cough, and weakness of 20 days' duration. Initial investigations suggested hepatic abscesses on ultrasonography. However, further evaluation with a contrast-enhanced computed tomography scan revealed a pancreatic uncinate process mass with multiple hepatic and skeletal metastases, suggestive of advanced pancreatic malignancy. Positron-emission tomography computed tomography confirmed the presence of a pancreatic mass with widespread metastatic lesions. Increased serum cancer antigen (CA) 19-9 and CA-125 levels also supported the diagnosis of metastatic pancreatic adenocarcinoma. This case highlights the diagnostic challenge of pancreatic carcinoma presenting atypically as fever with incidentally detected liver abscess-like lesions.

## Introduction

Pancreatic adenocarcinoma remains a highly aggressive cancer, constituting the vast majority of pancreatic malignancies and contributing substantially to global cancer mortality. Due to its subtle early course and deep retroperitoneal location, the disease usually escapes detection until it has reached an advanced or disseminated stage, which occurs in more than four of five patients. While patients most often come to attention with obstructive jaundice, unexplained weight loss, abdominal discomfort, or loss of appetite, symptoms in some individuals may manifest unusually. Presentations dominated by persistent fever or generalized inflammatory symptoms, closely resembling infectious or autoimmune disorders, are exceptionally uncommon and can lead to significant delays in establishing the correct diagnosis.

Fever as the initial and predominant manifestation of pancreatic carcinoma is unusual. It poses a significant diagnostic challenge, particularly in tropical regions where infectious etiologies such as liver abscess, tuberculosis, or parasitic infections are more prevalent. Misdiagnosis of an infectious process may delay identification of the underlying malignancy and result in a loss of the therapeutic window [1].

A small number of cases describing pancreatic carcinoma presenting as fever of unknown origin (FUO) have been reported in the literature. Similar to previously described cases, patients often present with prolonged fever in the absence of classic symptoms such as jaundice or significant abdominal pain, leading to an initial suspicion of infectious etiologies. However, unlike prior reports where imaging findings were either subtle or nonspecific, our case demonstrated multiple hepatic lesions closely mimicking abscesses, creating a stronger diagnostic bias toward infection. This overlap further delayed recognition of the underlying malignancy and highlights the variability and diagnostic complexity of such atypical presentations [1,2].

We present a case of metastatic pancreatic adenocarcinoma that initially manifested as FUO and liver abscess-like lesions on ultrasonography, underscoring the importance of maintaining a high index of suspicion for malignancy in patients with atypical and nonresolving febrile illness [2].

## Case presentation

A 54-year-old man, with no known comorbidities, presented to our hospital with a history of persistent fever and dry cough for the past 20 days, associated with loss of appetite and generalized weakness. The fever was moderate to high-grade (up to 101.6°F), intermittent, predominantly evening-rising, and associated with chills and malaise. It was partially relieved with antipyretics but showed no sustained response to antibiotic therapy. The patient reported reduced functional capacity over the preceding weeks, with increasing fatigue and decreased oral intake, although no significant weight loss was documented.

The patient denied any history of jaundice, abdominal pain, nausea, vomiting, diarrhea, urinary complaints, or significant weight loss. There was no history of recent travel, animal exposure, or tuberculosis contact. He was evaluated at a local hospital two weeks prior, where he was diagnosed with a lower respiratory tract infection and treated with intravenous antibiotics and antipyretics.

On admission, the patient appeared ill, febrile, and mildly dyspneic. His vital parameters were body temperature 101.6°F, elevated pulse rate 104 beats/minute, elevated respiratory rate 22 breaths/minute, blood pressure 118/76 mmHg, and oxygen saturation 96% on room air. There was no pallor, icterus, cyanosis, clubbing, or pedal edema. No peripheral lymphadenopathy or skin lesions were observed.

Systemic examination revealed mild hepatomegaly with a soft, tender liver edge palpable 3 cm below the right costal margin. There was no splenomegaly or free fluid. Respiratory examination revealed mild bibasal crepitations without added sounds. Cardiovascular and neurological examinations were normal.

Given the prolonged febrile illness with hepatomegaly and nonresolving symptoms despite antibiotic therapy, a provisional diagnosis of FUO with possible hepatic or systemic pathology was considered.

Routine laboratory investigations, including complete blood count, renal function tests, serum calcium, phosphorus, and uric acid, were within normal limits. Additional investigations, including erythrocyte sedimentation rate, C-reactive protein, and liver function tests, were also conducted. Two-dimensional echocardiography was normal. Stool examination and culture were unremarkable. Routine laboratory investigations revealed elevated inflammatory markers, mildly elevated total bilirubin, alkaline phosphatase, and transaminases suggestive of mild cholestatic involvement (Table 1).

**Table 1:** Summary of hematological and biochemical investigations of the patient AST: aspartate aminotransferase; ALT: alanine aminotransferase; ALP: alkaline phosphatase Reference ranges were obtained from standard laboratory reference ranges [3]

Parameter	Result	Reference range	Interpretation	Units
Erythrocyte sedimentation rate	35	<15	Elevated: inflammatory pattern	mm/hour
C-reactive protein	90	<10	Markedly elevated: inflammatory/malignant pattern	mg/L
Total bilirubin	1.4	0.3-1.0	Mildly elevated: cholestatic pattern	mg/dL
Direct bilirubin	0.8	0.1-0.3	Elevated	mg/dL
AST	43	0-35	Mildly elevated	U/L
ALT	42	4-36	Mildly elevated	U/L
ALP	253	30-120	Elevated: cholestatic pattern	U/L
Serum total protein	5.9	6.4-8.3	Low	g/dL
Serum albumin	3.0	3.5-5	Low: chronic disease	g/dL

To exclude infective causes, serological tests for dengue, malaria, HIV, and hepatitis B surface antigen were negative. Blood cultures were sterile after 72 hours of incubation. A chest radiograph showed no consolidation, cavitation, or hilar lymphadenopathy.

As the fever persisted despite broad-spectrum antibiotic coverage for presumed lower respiratory tract infection and no infective etiology was identified, further evaluation was planned to rule out occult abscesses, granulomatous disease, or malignancy.

**Figure 1:**
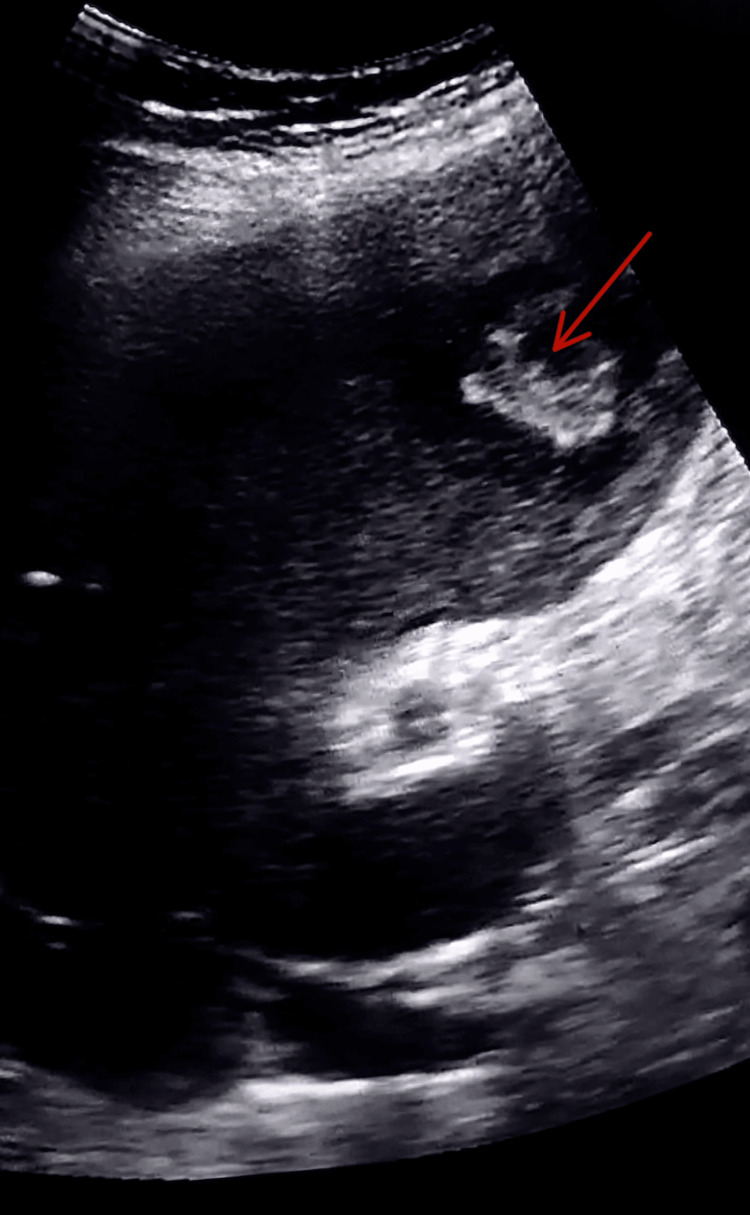
Ultrasonography of the abdomen showing multiple hyperechoic lesions in both lobes of the liver with hepatomegaly suggestive of abscess-like lesions

Ultrasonography revealed hepatomegaly (17.8 cm) with multiple well-defined, heterogeneous, predominantly hyperechoic lesions in both lobes of the liver, the largest measuring 39 × 30 mm in the left lobe. Gallbladder wall edema and mild hepatosplenomegaly were also noted (Figure 1).

The initial findings indicated the presence of multiple hepatic abscesses, prompting the initiation of empirical metronidazole therapy for the patient. However, the fever persisted, and the clinical condition remained unchanged.

**Figure 2:**
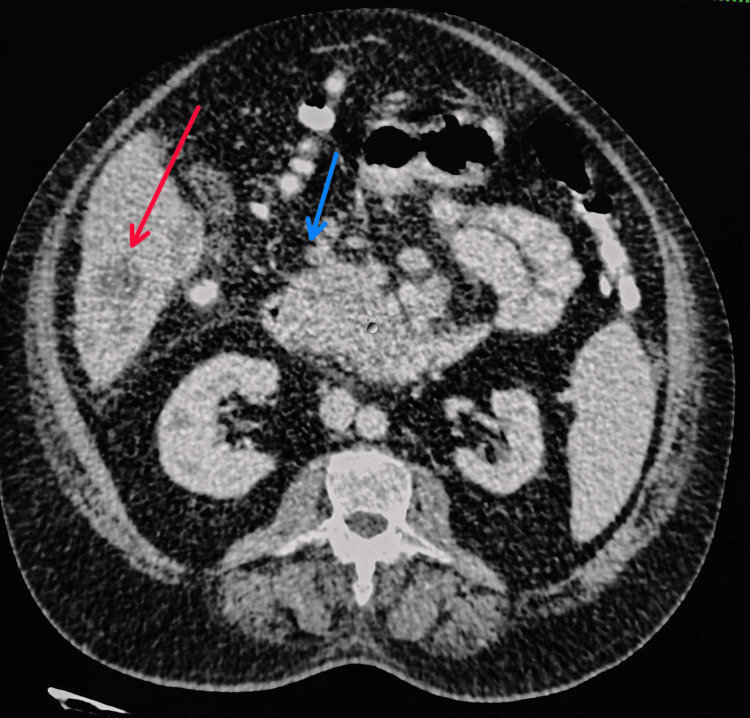
CECT abdomen showing multiple peripherally enhancing hepatic lesions (red arrow) and a hypodense pancreatic uncinate lesion (blue arrow) CECT: contrast-enhanced computed tomography

Contrast-enhanced computed tomography (CECT) of the abdomen and pelvis revealed multiple peripherally enhancing hepatic lesions with subcapsular collection, an ill-defined hypodense lesion in the uncinate process of the pancreas, and multiple lytic lesions in the bilateral pubic rami, left ileum, L3 vertebra, and left 5th rib. Hepatosplenomegaly and a mild right pleural effusion were also observed (Figure 2).

The overall imaging picture raised the possibility of an underlying neoplastic etiology, so a serum tumor marker profile was ordered along with serum lipase levels, and a positron-emission tomography computed tomography (PET-CT) scan was advised for further characterization (Table 2).

**Table 2:** Serum tumor marker profile CA 19-9: cancer antigen 19-9; CA-125: cancer antigen 125; AFP: alpha fetoprotein Reference ranges were based on standard laboratory reference values [4]

Parameter	Result	Reference range	Interpretation	Units
CA 19-9	508 U/mL	<37	↑ Elevated	U/mL
CA-125	6,230 U/mL	<35	↑ Significantly elevated	U/mL
AFP	3 ng/mL	<10	Normal	ng/mL

**Figure 3:**
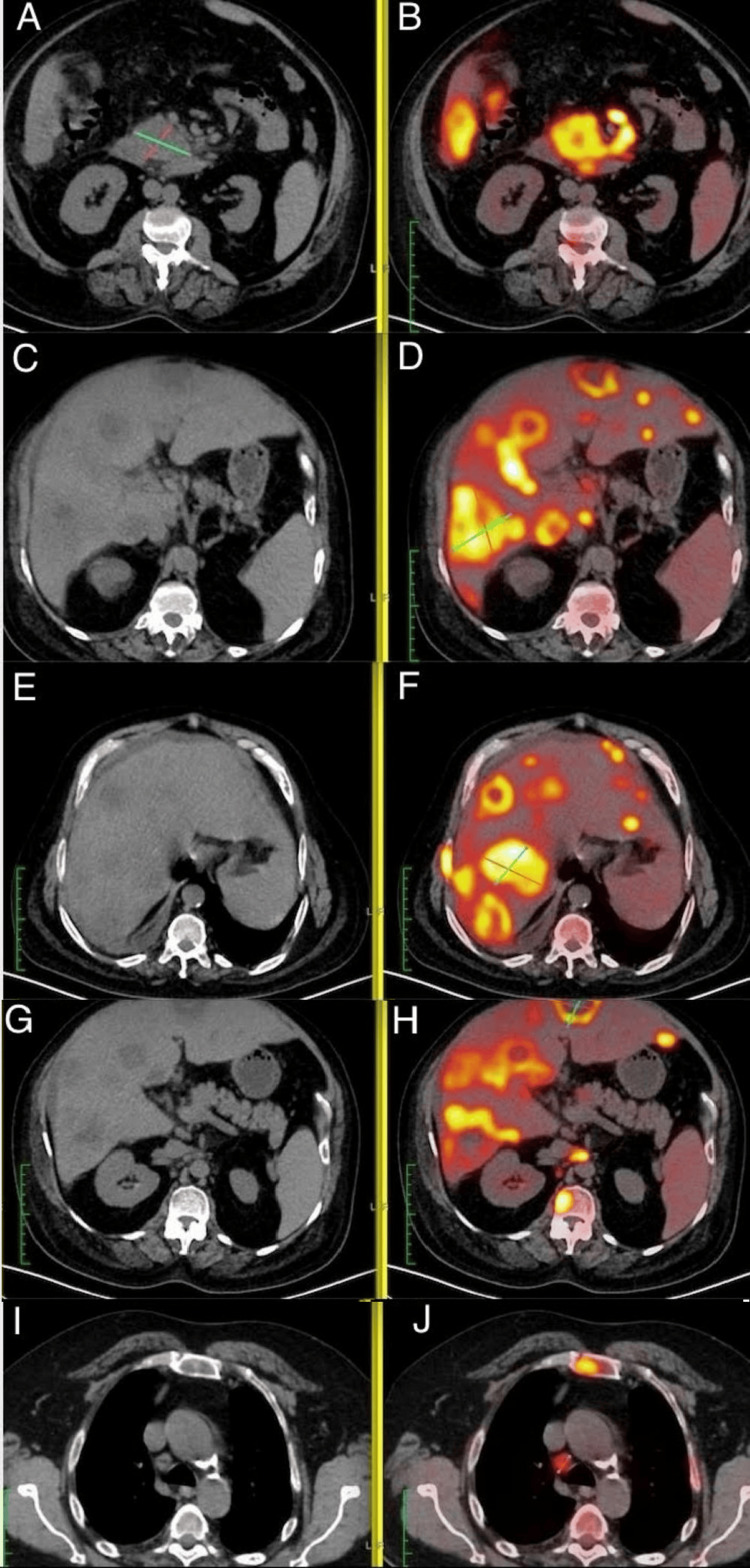
PET-CT demonstrating an FDG-avid lesion in the pancreatic uncinate process (A,B), multiple hypermetabolic hepatic lesions (C-H), and focal skeletal uptake (I,J), consistent with widespread metastatic disease PET-CT: positron-emission tomography computed tomography; FDG: fluorodeoxyglucose

The serum lipase level was 303 U/L, which was slightly elevated and pointed to possible pancreatic involvement. PET-CT demonstrated a fluorodeoxyglucose (FDG)-avid mass in the uncinate process of the pancreas measuring 53 × 86 mm with a standardized uptake value of 13.8, consistent with a primary pancreatic neoplasm. Multiple FDG-avid hepatic lesions with central necrosis were noted, with a maximum standardized uptake value of up to 21.0, along with mediastinal and abdominal lymphadenopathy, mild ascites, and right pleural effusion. Additionally, multiple FDG-avid skeletal metastases involving the clavicle, ribs, vertebrae, pelvis, and femur confirmed disseminated metastatic disease (Figure 3).

A confirmatory biopsy was not performed due to the patient’s clinical condition and extensive disease burden. The diagnosis was based on a combination of radiological findings, markedly elevated tumor markers, and the overall clinical picture, which strongly suggested advanced pancreatic adenocarcinoma. However, the absence of histopathological confirmation remains a limitation.

## Discussion

Pancreatic adenocarcinoma typically presents with local or systemic manifestations related to tumor invasion, biliary obstruction, or metastasis. The most common symptoms include epigastric pain radiating to the back, jaundice, and weight loss. In contrast, this patient's presentation with prolonged fever and generalized weakness led to an initial clinical impression of infection.

A limited number of cases describing pancreatic adenocarcinoma presenting as FUO have been reported in the literature. Similar to previously published reports, our patient presented with prolonged fever and absence of classic symptoms such as jaundice or abdominal pain. However, unlike most cases, the presence of multiple hepatic lesions initially mimicking abscesses created a stronger bias toward an infectious etiology, particularly in a tropical setting. This overlap contributed to diagnostic delay and highlights the variability in clinical presentation and imaging findings in such cases.

Fever may present as a paraneoplastic or secondary manifestation of pancreatic malignancy and may result from several mechanisms: tumor necrosis or local inflammation due to hepatic metastases, release of pyrogenic cytokines such as IL-1, IL-6, and tumor necrosis factor-α from the tumor or host immune cells, and secondary infection due to tumor necrosis or biliary obstruction [5].

In our patient, sterile blood cultures, absence of an infective focus, and lack of response to broad-spectrum antibiotics suggested a noninfectious origin of the fever, likely secondary to tumor-related inflammation.

The ultrasonographic appearance of multiple hyperechoic liver lesions initially suggested multiple hepatic abscesses, a common differential diagnosis in patients with fever and hepatomegaly in endemic areas. However, failure to respond to antimicrobial therapy and persistently elevated tumor markers (cancer antigen (CA) 19-9 and CA-125) raised suspicion of an underlying malignancy [6].

CECT findings of peripherally enhancing lesions with central necrosis and PET-CT evidence of high FDG uptake supported a diagnosis of metastatic deposits rather than infective abscesses. The presence of skeletal lesions further consolidated the neoplastic etiology [7].

The markedly elevated CA-125 level (6,230 U/mL) observed in this case is unusual and may reflect extensive metastatic disease, possible peritoneal involvement, or a heightened inflammatory response. While CA 19-9 is a more established marker for pancreatic adenocarcinoma, both markers lack absolute specificity and should be interpreted in conjunction with imaging and clinical findings. Mild lipase elevation may occur due to obstruction of the pancreatic duct or associated inflammation [3].

CA 19-9 is a well-established marker for pancreatic adenocarcinoma, with reported sensitivity of 80%-90% in advanced disease. Its elevation, along with markedly elevated CA-125, correlated with widespread metastatic involvement in this case [8].

PET-CT played a crucial role in identifying the primary pancreatic lesion, the extent of metastases, and differentiating malignant from infectious lesions. This imaging modality has become increasingly essential for evaluating an FUO when conventional imaging is inconclusive.

At presentation, the main differentials included pyogenic or amebic liver abscess, disseminated tuberculosis, hepatocellular carcinoma with necrosis, and metastatic malignancy of unknown primary origin.

The absence of microbiological evidence, nonresponse to antibiotics, normal alpha fetoprotein, and the PET-CT pattern eventually favored pancreatic adenocarcinoma with hepatic metastases [9,10].

An important diagnostic challenge in this case was differentiating hepatic abscesses from metastatic lesions. While both may present with fever and liver lesions, certain features help distinguish them. Hepatic abscesses are typically associated with leukocytosis, positive cultures, and clinical improvement with antimicrobial therapy. In contrast, metastatic lesions often show poor response to antibiotics, may demonstrate high FDG uptake on PET imaging, and are frequently associated with additional systemic findings such as lymphadenopathy or skeletal lesions. In our case, lack of microbiological evidence, nonresponse to antibiotics, and PET-CT findings strongly favored a malignant etiology.

Pancreatic adenocarcinoma has a poor prognosis, with a five-year survival rate below 10%. For metastatic disease, treatment is primarily palliative, focusing on symptom control and quality of life. Chemotherapy with gemcitabine-based regimens or FOLFIRINOX may be considered in patients with good performance status [11].

In this case, extensive metastases and rapid clinical deterioration precluded oncologic therapy, and the patient was managed with supportive and palliative measures. This case emphasizes that persistent fever unresponsive to antibiotics, especially when accompanied by hepatic lesions, should prompt evaluation for malignancy, even in the absence of typical cancer-related symptoms [12].

A key limitation of this case is the absence of histopathological confirmation. Although the diagnosis was strongly supported by imaging, tumor markers, and clinical progression, it remains a probable diagnosis rather than definitive. This limitation should be considered when interpreting the findings.

This case highlights several important clinical takeaways: persistent fever unresponsive to appropriate antimicrobial therapy should prompt reconsideration of noninfectious causes including malignancy, hepatic lesions in the setting of FUO should not be presumed to be abscesses without adequate response to treatment, advanced imaging modalities such as PET-CT can play a crucial role in differentiating infectious from malignant etiologies, and atypical presentations of pancreatic cancer, although rare, should be considered in prolonged undiagnosed febrile illness.

## Conclusions

Pancreatic adenocarcinoma rarely presents with prolonged fever and liver lesions mimicking abscesses, leading to diagnostic confusion. Persistent fever associated with hepatic lesions should raise suspicion for malignant etiologies when microbiological investigations are negative, and there is poor response to antimicrobial therapy. This case underscores the importance of maintaining a broad differential diagnosis and of utilizing advanced imaging modalities, such as PET-CT, to evaluate nonresolving febrile illnesses. While the diagnosis in this case was strongly supported by clinical and radiological evidence, the absence of histopathological confirmation remains a limitation. Early suspicion and recognition of such atypical presentations are vital for timely diagnosis, appropriate management, and improved patient outcomes, even when curative options are limited.
